# Increased risk of benign paroxysmal positional vertigo in patients with anxiety disorders: a nationwide population-based retrospective cohort study

**DOI:** 10.1186/s12888-016-0950-2

**Published:** 2016-07-15

**Authors:** Zi-Jun Chen, Cheng-Ho Chang, Li-Yu Hu, Ming-Shium Tu, Ti Lu, Pan-Ming Chen, Cheng-Che Shen

**Affiliations:** Department of Family Medicine, Kaohsiung Veterans General Hospital, Kaohsiung, Taiwan; Department of Psychiatry, Kaohsiung Veterans General Hospital, Kaohsiung, Taiwan; Division of Psychiatry, Faculty of Medicine, National Yang-Ming University, Taipei, Taiwan; Department of Psychiatry, Yuanshan & Suao Branch, Taipei Veterans General Hospital, No. 301, Sec. 1, Subin Rd., Suao Township, Yilan County 27047 Taiwan; Department of Psychiatry, Chiayi Branch, Taichung Veterans General Hospital, No.600, Sec. 2, Shixian Rd., West District, Chiayi City, 60090 Taiwan; Department of information management, National Chung-Cheng University, Chiayi, Taiwan

**Keywords:** Anxiety disorder, Benign peripheral persistent vertigo, Risk factor

## Abstract

**Background:**

The objective of this study was to evaluate the risk of benign peripheral persistent vertigo (BPPV) among patients with anxiety disorders by using the Taiwan National Health Insurance Research Database (NHIRD).

**Methods:**

We conducted a retrospective study of 15,470 participants (7735 anxiety disorder patients and 7735 control patients) selected from the NHIRD. Patients were observed for a maximum of 9 years to determine the rates of newly diagnosed BPPV. A Cox regression model was used to evaluate the risk of BPPV among the patients with anxiety disorders.

**Results:**

During the 9-year follow-up period, 178 (2.05 per 1000 person-years) anxiety disorder patients and 71 (0.81 per 1000 person-years) control patients were diagnosed with BPPV. The incidence risk ratio of BPPV between anxiety disorder patients and control patients was 2.52 (95 % confidence interval [CI], 1.90–3.37, *P* < .001). After adjustment for age, sex, and comorbidities, patients with anxiety disorders were found to be 2.17 times more likely to develop BPPV (95 % CI, 1.63–2.90, *P* < .001) than the control patients. Furthermore, female sex (HR = 1.81, 95 % CI, 1.31–2.50, *P* < .001) and cerebrovascular disease (HR = 1.53, 95 % CI, 1.00–2.34, *P* = .050) were independent risk factors for developing new-onset BPPV in patients with anxiety disorders.

**Conclusions:**

Anxiety disorder patients may have an increased risk of developing BPPV, especially those who are female or have cerebrovascular disease.

## Background

Anxiety disorders are the most prevalent mental disorders. Various population surveys have shown substantial discrepancies in prevalence rates. According to the European Study of the Epidemiology of Mental Disorders [1, 2], as many as 33.7 % of people are affected by an anxiety disorder during their lifetime, among whom generalized anxiety disorder accounts for 6.2 % and panic disorder 5.2 %. There is a consistent finding that the prevalence of anxiety disorders occurs roughly twice as often in females as in males [1, 3]. Anxiety disorders cause significant quality-of-life impairment [4] and are associated with immense health care costs [1, 5].

Benign paroxysmal positional vertigo (BPPV) is the most common type of peripheral vertigo [6] and is generally associated with nausea and vomiting. The symptoms are caused by the otoliths’ detaching from the macula and entering the semicircular canals [6]. BPPV attacks may be related to head trauma, a prolonged recumbent position, and various pathological processes such as inflammation of the inner ears. According to previous studies, the presence of a systemic disease, including hypertension, diabetes, high cholesterol, cerebrovascular diseases, and autoimmune diseases such as allergies and thyroid autoimmunity, may worsen the status of the posterior labyrinth, causing a more frequent otolith detachment and, in turn, BPPV [7–10]. However, in most BPPV cases, the etiology is idiopathic [11]. Patients typically present with frequent spontaneous remissions and recurrences. The recurrence rate per year is approximately 15 % [12]. Additionally, patients with BPPV are at higher risks of falls and impairment in the performance of daily activities [13]. Previous studies have reported that the prevalence of BPPV is between 10.7 and 64.0 cases per 100,000 persons, with a lifetime prevalence of 2.4 % [14]. In addition, epidemiological studies have demonstrated that the prevalence of idiopathic BPPV is higher among women and older adults of both sexes, with the peak onset between 50 and 60 years of age and a female-to-male ratio between 2:1 and 3:1 [14, 15].

Although vertigo is a common somatic complaint among patients with anxiety disorders, to our knowledge, only one case-controlled study has investigated the association between anxiety and subsequent BPPV. The results of the previous study indicated that anxiety disorders may be considered a precursor of BPPV [16]. However, the sample size of the case-controlled study was small, with 100 participants enrolled. In addition, the study used a self-reported rating scale for evaluating the severity of anxiety rather than diagnosis by a psychiatrist, which may have limited the clinical impact and its generalizability.

Several studies have reported an increased incidence of vestibular dysfunction among patients with anxiety disorders. In addition, evidence shows that vestibular dysfunction is considered one of the pathogeneses for BPPV. Moreover, numerous studies have confirmed the neural inflammation and impaired neuroendocrine response in patients with anxiety, and both of the similar inflammatory processes and neuroendocrine dysfunction are regarded as crucial elements in the pathophysiology of BPPV. Therefore, we hypothesized that anxiety disorders may play crucial roles in the development of subsequent BPPV. In response to the lack of national data in Asian areas and few longitudinal studies concerning the association between anxiety disorders and the risk of BPPV, we designed a nationwide population-based study to investigate the incidence of new-onset BPPV among patients with anxiety disorders. Furthermore, possible independent risk factors including hypertension, diabetes mellitus, hyperlipidemia, cerebrovascular disease, autoimmune diseases, and other possible systemic diseases for BPPV among patients with anxiety disorders were identified and analyzed.

## Methods

### Data sources

In 1995, Taiwan launched the NHI program, which covers 99 % of Taiwan’s population [17]. The NHI program offers universal medical care, including outpatient, inpatient, and emergency care, as well as traditional Chinese medicinal treatment. The NHI research database (NHIRD) contains comprehensive information regarding clinical visits, including prescription details and diagnostic codes based on the International Classification of Diseases, Ninth Revision, Clinical Modification (ICD-9-CM). The NHIRD is managed by the National Health Research Institutes (NHRI), and confidentiality is maintained according to the directives of the Bureau of the NHI [18]. The data source for our study was the Longitudinal Health Insurance Database 2000 (LHID 2000), which is a data set of the NHIRD. Data for the LHID were collected through both systematic and random sampling from the NHIRD; the database comprised data on one million individuals. The NHRI claims data indicated no significant differences in sex and age distribution or in average insured payroll-related amounts between the patients in the LHID and those in the original NHIRD [18].

### Study population

A retrospective cohort study was conducted using data extracted from the LHID 2000. Patients aged 20 years and older who were newly diagnosed with anxiety disorders between January 1, 2000, and December 31, 2004, were enrolled. The term “anxiety disorders” was defined in accordance with ICD-9-CM codes 300.0, 300.2, and 308.3. To ensure the accuracy of anxiety disorder diagnosis, only patients who were diagnosed with anxiety disorders by a psychiatrist were selected. In both the anxiety disorder and control groups, we excluded patients who were diagnosed with BPPV (ICD-9-CM code 386.11) prior to the enrollment date. Finally, we identified 7735 patients with anxiety disorders. To assemble a comparison cohort, we randomly selected 7735 enrollees without a history of anxiety disorders. These controls were matched with the study cohort at a ratio of 1:1 by age and sex according to the same exclusion criteria during the same period. To increase the diagnosis validity, only neurologist-diagnosed BPPV or otorhinolaryngologist-diagnosed BPPV was included as our primary clinical outcome. The participants in both the study and control cohorts were observed until being diagnosed with BPPV by a neurologist or an otorhinolaryngologist, death, withdrawal from the NHI system, or December 31, 2013.

### Statistical analysis

The incidence of newly diagnosed BPPV in the anxiety disorder and control patients was calculated and stratified by sex and age (older or younger than 60 years). Independent *t* tests and chi-square tests were used to examine the differences in the demographic characteristics between the anxiety disorder and control patients.

For each patient diagnosed with BPPV who was included in the study, the relevant period for calculating person-years at risk began on the enrollment date and ended on the date of BPPV diagnosis by a neurologist or an otorhinolaryngologist, death, withdrawal from the NHI system, or December 31, 2013. The same calculation was used for each person in the control cohort.

In our study, in addition to investigating whether patients with anxiety disorders have a higher risk of developing subsequent BPPV than that of control patients, we sought to identify the variables that are potential predictive factors for developing BPPV among patients with anxiety disorders. Therefore, a Cox proportional hazards regression model was employed twice: once for identifying possible confounding variables and excluding their effects on the process of evaluating whether anxiety disorders increase the risk of subsequent BPPV (Table 3), and once for attempting to identify the variables that may indicate subsequent BPPV among patients with anxiety disorders (Table 4). The variables used in the Cox model were age, sex, and common comorbidities, including hypertension, diabetes mellitus, chronic liver disease, autoimmune disease, congestive heart failure, hyperlipidemia, nephropathy, cerebrovascular disease, and chronic obstructive pulmonary disease (COPD). The variables that demonstrated a moderately significant statistical relationship with BPPV in the univariate analysis (*P* < .1) were entered through forward selection in a multivariate analysis.

## Results

### Participant selection

The sample comprised 7735 anxiety disorder patients and 7735 control patients without anxiety disorders. Among both cohorts, 58.3 % were female. The median age of both cohorts on the enrollment date was 43 years (interquartile range [IQR], 33–55 years), and the median follow-up period was 11.24 years (IQR, 9.97–12.61 years) for the anxiety disorder patients and 11.27 years (IQR, 10.00–12.64 years) for the control patients. Comorbidities, including hypertension, diabetes mellitus, chronic liver disease, autoimmune disease, congestive heart failure, hyperlipidemia, nephropathy, cerebrovascular disease, and COPD, were more common in the anxiety disorder cohort than in the control cohort. A comparison of the demographic and clinical variables between the anxiety disorder and control patients is presented in Table 1.

**Table 1:** Baseline characteristics of patients with and without anxiety disorders

Demographic data	Patients with anxiety disorders		Patients without anxiety disorders		*P* value
	*n* = 7735		*n* = 7735		
	*n*	%	*n*	%	
Age (years)^a^	43 (33–55)		43 (33–55)		
≥ 60	1417	18.3	1417	18.3	>.999
< 60	6318	81.7	6318	81.7	
Sex					
Male	3225	41.7	3225	41.7	>.999
Female	4510	58.3	4510	58.3	
Comorbidities					
Hypertension	1932	25.0	966	12.5	<.001
Diabetes mellitus	358	4.6	230	3.0	<.001
Chronic liver diseases	2314	29.9	857	11.1	<.001
Autoimmune diseases	679	8.8	311	4.0	<.001
Congestive heart failure	234	3.0	101	1.3	<.001
Hyperlipidemia	1411	18.0	620	8.0	<.001
Nephropathy	696	9.0	274	3.5	<.001
Cerebrovascular disease	792	10.2	279	3.6	<.001
COPD	1682	21.7	779	10.1	<.001
Follow-up years^a^	11.24 (9.97–12.61)		11.27 (10.00–12.64)		.012

^a^Median (interquartile range); COPD indicates chronic obstructive pulmonary disease

### Incidence rate of BPPV

During the study period, 178 (2.05 per 1000 person-years) patients with anxiety disorders and 71 (0.81 per 1000 person-years) control patients were diagnosed with BPPV. The incidence risk ratio (IRR) of BPPV between the anxiety disorder patients and control patients was 2.52 (95 % confidence interval [CI], 1.90–3.37, *P* < .001). When stratified by sex and age, the IRR of BPPV was still higher in the patients with anxiety disorders than in the control patients. The results are shown in Table 2.

**Table 2:** Incidence of benign paroxysmal positional vertigo in patients with and without anxiety disorders

	Patients with anxiety disorders		Patients without anxiety disorders		Risk ratio (95 % CI)	*P* value
	No. of BPPV	Per 1000 person-years	No. of BPPV	Per 1000 person-years		
Total	178	2.05	71	0.81	2.52 (1.90–3.37)	<.001
Age						
≥ 60	45	2.82	16	0.99	2.85 (1.58–5.39)	<.001
< 60	133	1.87	55	0.77	2.43 (1.76–3.39)	<.001
Sex						
Male	52	1.43	25	0.68	2.09 (1.27–3.51)	.002
Female	126	2.49	46	0.90	2.76 (1.95–3.96)	<.001

*CI* indicates confidence interval

**Fig. 1:**
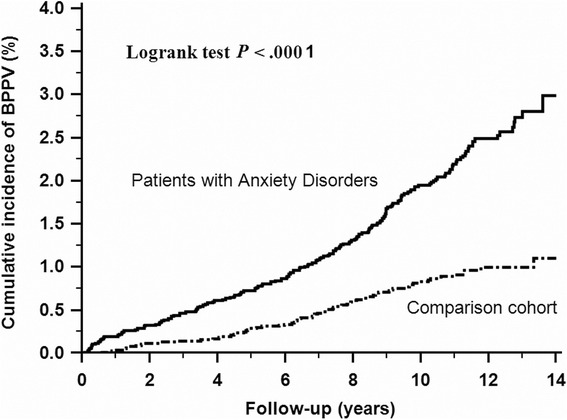
Cumulative incidence of benign paroxysmal positional vertigo in anxiety disorders and comparison cohort. The cumulative incidence of benign paroxysmal positional vertigo in patients with anxiety disorders was significantly higher than that in the comparison cohort

The cumulative incidence of BPPV in the patients with anxiety disorders was significantly higher than that in the control cohort (log-rank test, *P* < .001, Fig. 1).

### Effects of anxiety disorders on risk of BPPV

After adjustment for age, sex, and comorbidities, the hazard ratio (HR) for developing BPPV after anxiety disorder diagnosis during the follow-up period was 2.17 times (95 % CI, 1.63–2.90, *P* < .001) greater than that of the control cohort (Table 3).

**Table 3:** Analyses of risk for benign paroxysmal positional vertigo in patients with and without anxiety disorders

Predictive variables	Univariate analysis		Multivariate analysis	
	HR (95 % CI)	*P* value	HR (95 % CI)	*P* value
Anxiety disorders	2.52 (1.92–3.32)	<.001	2.17 (1.63–2.90)	<.001
Age (≥60 = 1, <60 = 0)	1.44 (1.08–1.92)	.014	1.09 (0.78–1.53)	.601
Sex (female = 1, male = 0)	1.60 (1.23–2.10)	.001	1.65 (1.26–2.17)	<.001
Comorbidities				
Hypertension	1.76 (1.34–2.32)	<.001	1.06 (0.75–1.48)	.751
Diabetes mellitus	1.93 (1.18–3.15)	.009	1.18 (0.70–1.99)	.528
Chronic liver diseases	1.48 (1.12–1.96)	.006	0.95 (0.70–1.29)	.719
Autoimmune diseases	1.28 (0.80–2.04)	.309		
Congestive heart failure	1.95 (1.04–3.67)	.039	1.02 (0.52–1.98)	.958
Hyperlipidemia	2.06 (1.53–2.76)	<.001	1.36 (0.96–1.92)	.084
Nephropathy	2.46 (1.72–3.54)	<.001	1.69 (1.14–2.48)	.008
Cerebrovascular disease	2.33 (1.64–3.32)	<.001	1.51 (1.02–2.24)	.039
COPD	1.87 (1.40–2.49)	<.001	1.35 (0.99–1.84)	.055

*HR* indicates hazard ratio, *CI* indicates confidence interval, *COPD* indicates chronic obstructive pulmonary disease

### Risk factors for BPPV in patients with anxiety disorders

We applied a univariate analysis to identify the possible risk factors for subsequent BPPV in the anxiety disorder cohort. The results are displayed in Table 4, demonstrating that older age (HR = 1.50, 95 % CI, 1.07–2.10, *P* = .019), female sex (HR = 1.74, 95 % CI, 1.26–2.41, *P* = .001), hypertension (HR = 1.56, 95 % CI, 1.14–2.12, *P* = .005), diabetes mellitus (HR = 1.99, 95 % CI, 1.17–3.37, *P* = .011), hyperlipidemia (HR = 3.74, 95 % CI, 1.67–8.38, *P* < .001), cerebrovascular disease (HR = 1.90, 95 % CI, 1.29–2.80, *P* = .001), and COPD (HR = 1.47, 95 % CI, 1.06–2.04, *P* = .021) are possible risk factors for subsequent BPPV. After the multivariate analysis, we confirmed that female sex (HR = 1.81, 95 % CI, 1.31–2.50, *P* < .001) and cerebrovascular disease (HR = 1.53, 95 % CI, 1.00–2.34, *P* = .050) were independent risk factors for subsequent BPPV development in the patients with anxiety disorders.

**Table 4:** Analyses of risk factors for benign paroxysmal positional vertigo in patients with anxiety disorders

Predictive variables	Univariate analysis		Multivariate analysis	
	HR (95 % CI)	*P* value	HR (95 % CI)	*P* value
Age (≥60 = 1, <60 = 0)	1.50 (1.07–2.10)	.019	1.13 (0.76–1.67)	.561
Sex (female = 1, male = 0)	1.74 (1.26–2.41)	.001	1.81 (1.31–2.50)	<.001
Comorbidities				
Hypertension	1.56 (1.14–2.12)	.005	1.18 (0.81–1.71)	.392
Diabetes mellitus	1.99 (1.17–3.37)	.011	1.47 (0.84–2.57)	.173
Chronic liver diseases	1.14 (0.84–1.57)	.403		
Autoimmune diseases	1.16 (0.70–1.91)	.566		
Congestive heart failure	1.55 (0.76–3.15)	.226		
Hyperlipidemia	3.74 (1.67–8.38)	<.001	1.24 (0.86–1.80)	.262
Nephropathy	1.40 (0.89–2.21)	.146		
Cerebrovascular disease	1.90 (1.29–2.80)	.001	1.53 (1.00–2.34)	.050
COPD	1.47 (1.06–2.04)	.021	1.27 (0.90–1.80)	.169

*HR* indicates hazard ratio, *CI* indicates confidence interval, *COPD* indicates chronic obstructive pulmonary disease

## Discussion

According to our thorough research, this is the first large nationwide population-based cohort study to compare the HRs for newly diagnosed BPPV in patients with and without anxiety disorders. The two major findings of this study are as follows. First, the risk of BPPV was significantly higher among the patients with anxiety disorders. Second, we suggest that female sex and cerebrovascular disease may be considered risk factors for developing subsequent BPPV among patients with anxiety disorders.

Psychiatric comorbidities in vestibular disorders, such as Meniere’s disease and vestibular neuronitis, have been broadly described [19–26]. Nevertheless, few studies have examined psychiatric disorders as precursors of BPPV. Monzani et al. investigated this concern by using a case-controlled study of 100 participants; the results showed that higher levels of anxiety may be considered a precursor of BPPV [16]. This finding was consistent with our results and reinforces the dependability of our study findings.

No direct pathophysiological link between anxiety disorders and BPPV has been established. We postulated several possible mechanisms for the increased risk of BPPV in patients with anxiety disorders. First, a higher prevalence of vestibular dysfunction in anxiety patients may result in BPPV development. The literature demonstrates that abnormal vestibular sensitivity occurs in anxiety patients [27], and vestibular dysfunction is regarded as one of the pathogeneses for BPPV [28, 29]. Second, an anxiety-disorder-related inflammatory response may result in subsequent BPPV development. Numerous studies have proposed a relationship between anxiety disorders and inflammatory processes [30–33], and neural inflammation is known to play a crucial role in the pathophysiology of BPPV [34, 35]. Third, an impaired neuroendocrine response induced by anxiety disorders may cause BPPV. Anxiety disorders are presumed to result in abnormal activation of the hypothalamus–pituitary–adrenal axis, which may induce dysfunction of neuroendocrine responses [36]. This neuroendocrine dysfunction results in inner ear blood flow imbalance [37], thus influencing otoconial homeostasis [16, 38], which has been considered a potential risk factor for developing BPPV [37].

Another finding of this study was that female sex and cerebrovascular disease may be risk factors for developing BPPV among patients with anxiety disorders. As mentioned previously, BPPV occurs more often in females than in males [14], with a female-to-male ratio of between 2:1 and 3:1, which may explain the results of our study. In addition, previous studies have advocated disruption of the anterior vestibular artery as an etiology of BPPV [39], which may explain why cerebrovascular disease is a risk factor for developing BPPV. Disruption of the anterior vestibular artery results in degeneration of the otolith, increasing the probability of the otolith’s floating into the semicircular canals, thereby promoting the development of BPPV [39]. Furthermore, evidence shows that microcirculatory damage is associated with sensory epithelial dysfunction in the inner ear, which exacerbates detachment of the otolith from the otolith membrane and impairs absorption of the otolith in vestibular dark cells, thereby inducing the onset of BPPV [10, 40].

The strengths of our study are the large sample size and the valid diagnosis of anxiety disorders and BPPV by experts in the claims database. Nevertheless, certain limitations of our findings should be considered. First, the NHIRD does not provide detailed information on several factors that may have been crucial confounding variables in our study, such as tobacco use, alcohol consumption, favored sleeping position, and family history of BPPV. In 2006, Sunami et al. reported that smoking and alcohol consumption were associated with a lower incidence of BPPV [41]. One study reported that an ear-down 45° head position during sleep may be an etiological factor for BPPV; possibly because this position makes it easier for detached otoconia to fall into the posterior or lateral semicircular canal [42]. In addition, a previous study revealed a familial tendency for the occurrence of BPPV [43]. Second, deviations in the coding of the claims data were inevitable. In our study, we attempted to achieve high validity by obtaining coding from a psychiatrist, neurologist, or otorhinolaryngologist. In addition, a matched control cohort was used to offset possible coding errors and minimize bias. However, thus far, there has been no validation study of the diagnosis and/or severity of anxiety disorders that involved using the NHIRD as a research database. Third, the prevalence of anxiety disorders and BPPV may be underestimated because only subjects seeking medical evaluation could be identified; however, this most likely resulted in underestimating the association between anxiety disorders and BPPV. Finally, information regarding the severity of anxiety disorders is lacking in the NHIRD; thus, we could not investigate the impact of the severity of anxiety disorders on BPPV in this study.

## Conclusions

Our nationwide population-based retrospective cohort study provides further evidence for an increased risk of BPPV in patients with anxiety disorders. Clinicians should be aware of the possibility of new-onset BPPV in anxiety disorder patients. Prospective studies, especially those with greater access to patient-level data, are warranted to confirm our findings.

## Abbreviations

BNHI, Bureau of National Health Insurance; BPPV, benign peripheral persistent vertigo; COPD, chronic obstructive pulmonary disease; ICD-9-CM, International Classification of Diseases, Ninth Revision, Clinical Modification; LHID, Longitudinal Health Insurance Database; NHI, National Health Insurance; NHIRD, National Health Insurance Research Database; NHRI, National Health Research Institutes
